# Mechanism of Microbiota-Gut-Brain in Perimenopausal Depression: An Inflammatory Perspective

**DOI:** 10.1017/erm.2025.10011

**Published:** 2025-09-04

**Authors:** Xia Yu, Yi Zuo, Yang Yang, Wei Cheng, Mingxiu Shi, Luona Cheng, Qixiang Shao, Yongjun Xu, Li Chen

**Affiliations:** 1 Fuqing City Hospital Affiliated to Fujian Medical University, Fuqing, P. R. China; 2 Department of Intensive Care Unit, Affiliated Huai’an No. 2 People’s Hospital of Xuzhou Medical University, Huai’an, P. R. China; 3 Fuzong General Teaching Hospital of Fujian University of Traditional Chinese Medicine, Fuzhou, P. R. China; 4 Institute of Medical Genetics and Reproductive Immunity, The Digestive and Reproductive System Cancers Precise Prevention Engineering Research Center of Jiangsu Province, School of Medical Science and Laboratory Medicine, Jiangsu College of Nursing, Huai’an, P. R. China; 5 Laboratory of Basic Medicine, 900th Hospital of PLA Joint Logistics Support Force, Fuzhou, P. R. China; 6 Fujian Provincial Key Laboratory of Transplant Biology, Fuzong Clinical Medical College of Fujian Medical University, Fuzhou, |P. R. China; 7 Fuzong Teaching Hospital of Fujian University of Traditional Chinese Medicine (900th Hospital), Fuzhou, P. R. China; 8 Laboratory of Basic Medicine, Dongfang Hospital of Xiamen University, School of Medicine, Xiamen University, Fuzhou, P. R. China; 9 Department of Neurosurgery, Fuzong Clinical Medical College of Fujian Medical University, Fuzhou, P. R. China

**Keywords:** gut microbiota, microbiota-gut-brain axis, perimenopausal depression, probiotics, neuroinflammation

## Abstract

**Background:**

Perimenopausal women often experience physiological and psychological decline due to the effects of oestrogen fluctuations and the decline of ovarian function, leading to significantly increased depression rates, decreases in the quality of life and mental health issues. Studies have shown that the gut microbiota exerts anti-perimenopausal depression (PMD) effects via the microbiota-gut-brain (MGB) axis, the mechanisms of which may be related to inflammation. In this review, we discuss the effects and mechanisms of gut microbiota in PMD and provide new insights for future PMD treatment.

**Methods:**

This review elaborates on the role of MGB axis in PMD from different aspects of inflammation, including gut microbiota metabolites, inflammatory signaling pathways, and clinical applications.

**Results:**

Disorders of gut microbiota and decreased levels of gut microbiota metabolites (short-chain fatty acids, monoamine neurotransmitters) may cause PMD. The mechanism of intestinal microbiota-mediated inflammation may be related to TLR4/NF-κB pathway, NOD-like receptor protein 3 (NLRP3) inflammasome pathway and JAK-STAT pathway. At the same time, it was found that gut microbiota (probiotics, prebiotics, etc.) had good therapeutic potential in the treatment of PMD.

**Conclusions:**

MGB axis mediated inflammation may play an important role in PMD. The application of gut microbiota in the treatment of PMD patients has profound clinical transformation value, but a lot of efforts are still needed.

## Introduction

**Figure 1.**
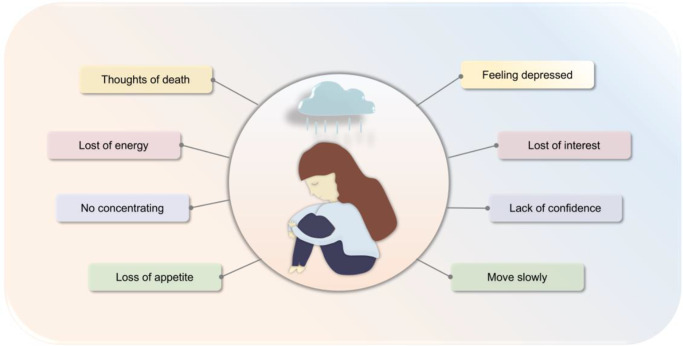
PMD signs and symptoms, including possible suicidal tendencies, sleep disorders, lack of confidence, low mood, low energy and other symptoms. PMD: perimenopausal depression.

Depression is a disorder with high incidence, high suicide and high disability rates (Ref. 1). Globally, more than 300 million individuals have depression, with a disease prevalence of 4.4% (Refs 2, 3). Depression has become the second major disease affecting human health. According to the World Health Organization, depression will be the leading global disease burden by 2030 (Ref. 4). Epidemiological survey data have shown that major depressive disorder (MDD) has obvious gender differences, with the lifetime prevalence of females exceeding 20%, which is twice that of males (Ref. 5). The perimenopausal period is the peak period of depression in women (Ref. 6), with approximately 1.5 million women entering the perimenopause each year (Refs 7, 8). Perimenopause is the time from declining ovarian function to the first year after menopause, usually occurring between the ages of 45 and 55 years. Women enter this perimenopausal stage due to a decline in ovarian function and hormone fluctuations, coupled with social, family, psychological and other pressure aspects, eventually leading to depression. Perimenopausal symptoms, often characterized by disturbed sleep, mood disturbances, decreased interest and low energy, may overlap with or complicate depressive symptoms (Figure 1). Studies have shown that women with a MDD history are prone to relapse during the perimenopause (Ref. 9). Of these, 45%–68% will suffer from depression aggravation, while the proportion of premenopausal women is 28%–31% (Refs 10, 11). Currently, perimenopausal depression (PMD) is mainly treated with oestrogen replacement therapy and 5-hydroxytryptamine (5-HT) reuptake inhibitors, with many studies confirming that improved oestrogen levels can relieve depressive symptoms (Refs 12, 13, 14). However, some studies have reported that depression symptoms in perimenopausal women become aggravated after stopping oestrogen (Ref. 15). Also, oestrogen replacement therapy for PMD has many side effects, which may lead to increased breast cancer, endometrial cancer, venous thrombosis and coronary heart disease risks (Refs 16, 17). Additionally, conventional drug therapy still has adverse reactions of slow onset and high recurrence rate, which brings a huge economic burden to patients. Therefore, it is urgent to further study the pathogenesis of perimenopausal depression and develop new treatment methods. With advancing research, the gut microbiota has come to prominence and is increasingly recognized in the microbiota-gut-brain (MGB) axis. The gut microbiota affects brain homeostasis via the MGB axis, and is not only involved in regulating circulating serotonin, kynurenine, tryptophan and short-chain fatty acids (SCFAs) availability, but also affects blood–brain barrier (BBB) permeability, peripheral immune system cell activation and brain microglial function (Refs 18, 19). An increasing number of studies have shown that the gut microbiota may affect brain function via neuroinflammatory pathways, thereby regulating behaviours such as anxiety and depression (Refs 20, 21, 22, 23). Currently, when compared with oestrogen therapy and antidepressant medication, gut microbiota therapy for depression has been shown to be relatively safe, with few side effects (Table 1). However, gut microbiota-mediated inflammation mechanisms in PMD are largely unexplored. To address this, in this review, we preliminarily discuss gut microbiota mechanisms and potential therapeutic prospects in PMD development via inflammatory pathways, and identify PMD prevention and treatment strategies.

**Table 1.** The advantages and disadvantages of oestrogen, antidepressants and the gut microbiota for treating PMD

Treatment	Action mechanism	Advantages	Disadvantages	References
Estrogen Therapy	Supplements exogenous oestrogen to regulate the neuroendocrine system, alleviating emotional disturbances caused by hormonal fluctuations during perimenopause.	Directly targets hormonal imbalances, effectively improving perimenopausal symptoms such as hot flashes and insomnia, and significantly regulating mood.	Long-term use increases breast cancer and thrombosis risks; not suitable for individuals with a history of hormone-dependent tumors. Efficacy varies depending on the administration route (oral versus transdermal).	(Refs 16, 17, 24–26)
Antidepressants (SSRIs/SNRIs)	Enhances neurotransmitter levels (5-HT, NE, DA) in the synaptic cleft to alleviate depressive symptoms.	Proven efficacy and suitable for patients with moderate to severe depression. Relatively rapid onset of action.	Numerous side effects (nausea, insomnia, etc.). Requires long-term medication, with a high relapse rate upon discontinuation.	(Refs 27–29)
Modulating the Gut Microbiota	Modulates gut microbiota composition (e.g., increasing *Lactobacillus* and *Bifidobacterium*), reduces inflammatory cytokines (IL–6, TNF-α), promotes short-chain fatty acid synthesis and improves gut-brain axis function.	High safety profiles with potential for metabolic and immune regulation; may reduce dependence on other treatments and improve overall health.	Efficacy varies significantly among individuals, with a slow onset of action. Lacks standardized protocols and limited clinical evidence.	(Refs 22, 30)

Abbreviations: 5-HT: 5-hydroxytryptamine; DA: dopamine; NE: norepinephrine; SSRIs: selective serotonin reuptake inhibitors; SNRIs: serotonin-norepinephrine reuptake inhibitors.

## The gut microbiota and PMD

The intestinal microecosystem is composed of intestinal epithelial cells, the gut microbiota and the intestinal mucosal immune system. Among these, the gut microbiota plays a key role in protecting the intestinal mucosal barrier. The human intestine contains trillions of microorganisms, including bacteria, viruses, archaea and fungi, collectively forming a microbial genome approximately 100–150 times larger than the human genome. The intestinal microbiome plays an important physiological role in food digestion, metabolism, intestinal barrier maintenance and immune system regulation (Ref. 31). The gut microbiota is mainly composed of *Firmicutes*, *Bacteroidetes*, *Actinobacteria* and *Proteobacteria*, with *Firmicutes* and *Bacteroidetes* accounting for 90% of intestinal microbes (Refs 31, 32).

At the phylum level, patients with depression usually show decreased abundance of *Firmicutes* and increased abundance of *Bacteroidetes*, *Proteobacteria* and *Actinobacteria.* The relative ratio of *Firmicutes* to *Bacteroidetes* (F/B) has been used as a measure of gut microbiota health. Clinical studies have shown that the F/B ratio decreases in MDD patients (Refs 33, 34). A cross-sectional study found significantly reduced Firmicutes abundance in MDD patients, which may lead to diminished SCFA production, potentially contributing to low-level inflammation in depression (Ref. 35). However, this study had limitations, including a small sample size and insufficient assessment of dietary factors (Ref. 35). Zhao et al. observed that postmenopausal women exhibited lower F/B ratios and reduced relative abundance of *Lachnospira* and *Roseburia* compared to premenopausal women (Ref. 36). Similarly, a Chinese metagenome-wide association study revealed decreased *Firmicutes* and *Roseburia spp.*, alongside increased *Bacteroidetes* and toluene-producing *Tolumonas*, in postmenopausal women (Ref. 37). However, some clinical studies have reported that the F/B ratio was increased in patients with MDD (Refs 38, 39, 40, 41). A Korean animal study also observed that ovariectomized (OVX) rats had increased F/B ratios and *Lachnospiraceae* and *Ruminococcaceae* family abundance while *Muribaculaceae* family abundance was decreased (Ref. 42). In addition, a meta-analysis showed that there was no difference in the abundance of *Bacteroidetes* and *Firmicutes* in patients with depression (Ref. 43). Meanwhile, in the subgroup meta-analysis, it was found that in patients with depression who had not taken psychotropic medications, the abundance of *Firmicutes* decreased while that of *Bacteroidetes* increased (Ref. 43). It was also found that dietary and regional differences could affect the composition of the gut microbiota (Ref. 43). Therefore, these differences may be attributed to several factors, such as clinical and demographic characteristics, sample differences, dietary patterns, regional differences, as well as medication use and other influencing factors (Table 2). In future research, firstly, strict experimental controls should be implemented, e.g., controlling diet (e.g., standardized dietary records), regional contexts (multicentre studies) and medication use (e.g., the exclusion of recent antibiotic/hormone therapy). Secondly, to verify mechanisms, animal models could be used to simulate different diet/hormone environments to clarify causal relationships between the F/B ratio and depressive phenotypes (e.g., germ-free mice transplanted with specific bacteria). Finally, standardized reporting should also be adopted; population characteristics and experimental methods should be better annotated, and confounding factors in the literature could be explored to help reduce data/results heterogeneity. Lim et al. reported that the relative abundance of beneficial intestinal bacteria (*Lactobacillus*, *Clostridium* and *Eubacterium*) in OVX rats was significantly reduced (Ref. 42). Li et al. also showed that the relative abundance of toxin-related *Cyanobacteria* in OVX rat intestines was increased when compared with a control group (Ref. 44). Huang et al. observed that PMD model mice had a reduced abundance of beneficial intestinal bacteria, such as *Lactobacillus*, *Alloprevotella*, *Akkermansia* and *Allobaculum*, as well as an increased abundance of harmful bacteria, such as *Muribaculaceae* (Ref. 45). Therefore, PMD occurrence may be related to decreased beneficial bacteria and increased harmful bacteria in the gut. Interestingly, two meta-analyses reported increased beneficial bacteria (*Lactobacillus*) abundance in patients with MDD (Refs 20, 21), indicating differential roles for different species in this genus, and suggesting no clear boundaries between beneficial and pathogenic bacteria; therefore, a gut microbiota balance appears to be more important than regulating a single bacterial class. These studies suggest that PMD occurrence may be related to a disordered gut microbiota, but specific mechanisms remain unclear. Additionally, a cluster analysis indicated that intestinal microflora composition in patients with depression in the US and China differed, with some microbial changes unique to patients in China, such as increased *Eggerthella* and *Acidaminococcus*, and decreased *Coprococcus*, *Fusicatenibacter* and *Prevotellaceae.* Such gut microbiota differences suggest that countries or geographical regions need to develop microbiome databases that are tailored to specific patient characteristics to guide future gut microbiome investigations (Ref. 20). However, few studies have examined gut microbiota differences in patients with PMD across different countries.

**Table 2.** F/B ratio differences and potential influencing factors

Influencing Factors	Impact Mechanism	Evidence	Explanation for Contradictory Results
1. Regional and Dietary Differences	Dietary structure: High-fiber diets promote *Bacteroidetes* proliferation, while high-fat/high-sugar diets may increase *Firmicutes* abundance. Regional characteristics: significant gut microbiota composition differences arise due to different dietary patterns between Eastern and Western populations.	- Chinese population (high-fiber/low-fat diet): lower F/B ratios with *Bacteroidetes* enrichment (Ref. 30); -Western population (high-fat/low-fiber diet): higher F/B ratios (Refs 53, 54).	Studies in China show that postmenopausal women have decreased F/B ratios (Ref. 37), possibly related to traditional Asian high-fiber diets; whereas Korean ovariectomized rats fed high-fat diets exhibit increased F/B ratios (Ref. 42).
2. Clinical and Demographic Characteristics	Menopausal stage: Differences in hormonal fluctuations between perimenopause and postmenopause; BMI and metabolic status: Higher *Firmicutes* abundance is typically observed in obese individuals.	- An abrupt drop in oestrogen levels at postmenopause may exacerbate gut dysbiosis (Ref. 7); - Obese MDD patients have significantly higher F/B ratios when compared to non-obese patients (Ref. 55).	Zhao et al. focused on lean Chinese postmenopausal women (Ref. 37), while Western MDD cohorts often include individuals with higher BMIs, which may amplify F/B differences (Refs 56–58).
3. Medication Interference	Antibiotics/hormone replacement therapy: directly alters gut microbiota composition. Antidepressants: SSRIs may inhibit *Firmicutes.*	- Antibiotic use reduces F/B ratios (Ref. 59). - Hormone replacement therapy increases *Lachnospiraceae* abundance (Ref. 60).	Some studies did not exclude medication interference, and increased F/B ratios may be related to uncontrolled hormone therapy or antidepressant use (Refs 61, 62).
4. Methodological Differences	Sequencing technology and data analysis: differences in resolution between 16S rRNA and metagenomics. Sample size: small sample studies are more susceptible to individual variability.	Metagenomics more accurately identifies species-level differences, while 16S rRNA may overestimate phylum-level changes (Refs 36,37,42,63–66).	Different sequencing technologies (Refs 36, 37) may lead to biases in species abundance assessments. Contradictory results can stem from insufficient statistical power (e.g., sample size <100) (Refs 67,68).

Abbreviations: BMI: body mass index; MDD: major depressive disorder; SSRIs: selective serotonin reuptake inhibitors.

**Figure 2.**
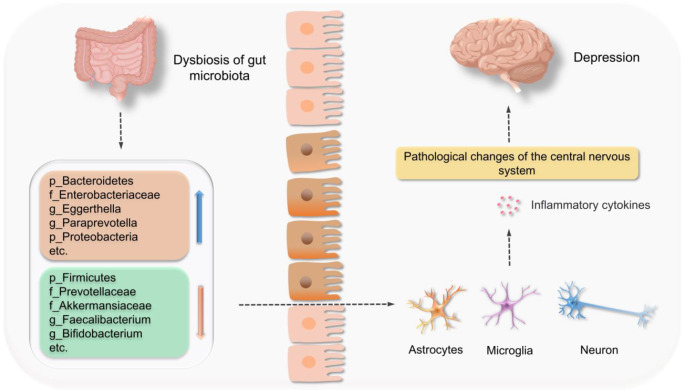
Associations between gut microbiota dysbiosis and pathological changes in the central nervous system (CNS) during depression. An imbalanced gut microbiota increases abundance in the *Bacteroidetes* phyla, the *Enterobacteriaceae* family, and the *Eggerthella* genus, and also decreases abundance of the *Firmicutes* phyla, and *Prevotellaceae* and *Akkermansiaceae* families. A disordered gut microbiota also leads to microglial activation and proinflammatory cytokine secretion (interleukin (IL)-1β, IL-6, etc.), which promotes astrocyte activation, causes neuronal damage (Refs 51,52) and neuroinflammation in the CNS, and eventually depression.

When compared to healthy individuals, a gut microbiota disorder occurs in patients with depression (Figure 2). Although data from recent studies are not very consistent, one common feature is increased proinflammatory and decreased anti-inflammatory bacteria (Refs 20, 21, 22). Among these, proinflammatory bacteria include *Alistipes*, *Eggerthella*, *Flavonifractor*, etc., while anti-inflammatory bacteria include *Bifidobacterium spp.*, *Coprococcus*, *Eucbacterium*, *Eubacterium rectale*, *Fecalibacterium*, *Fecalibacterium prausnitzii*, *Lactobacillus spp.*, *Prevotella* and *Roseburia*, amongst others (Ref. 19). A disturbed gut microbiota can lead to microglial activation and cause neuroinflammation in the central nervous system (CNS). Microglias are resident immune cells in the brain and have key roles in different neurodevelopmental processes for normal brain maturation and function, such as neurogenesis, synapse shaping and defences against infection. Resting microglia maintain homeostasis via regulated cytokines, which are secreted by astrocytes and other cells. Critically, microglia and astrocyte structure and function changes have been implicated in depression (Refs 46, 47). Studies have shown that germ-free (GF) mice exhibited global microglial defects, with altered cell proportions (Refs 48, 49) and immature phenotypes, leading to impaired innate immune responses (Ref. 48). Wei et al. observed that butyrate (a gut microbiota metabolite) supplementation improved chronic alcoholic CNS injury by inhibiting microglia-mediated neuroinflammation via GPR109A/peroxisome proliferator-activated receptor gamma (PPAR-γ)/toll-like receptor (TLR) 4-nuclear factor-κB (NF-κB) signalling (Ref. 50). Furthermore, GF mouse microglia engraftment of change in the form of time and gender specificity. In adult mice, Thion et al. observed that GF female microglia exhibited dysregulated genes related to cell morphogenesis, transcriptional regulation, adaptive immune responses, cell migration and chemotaxis, whereas GF male microglia showed no major changes (Ref. 49). Thus, PMD caused by a disordered gut microbiota may be related to inflammation.

## The MGB axis

In recent years, many studies have reported bidirectional communication signal pathways between the gut microbiota and the brain, also known as the MGB axis (Refs 22, 69). This axis comprises the gut, the gut microbiota and the nervous system, collectively referred to as the ‘second brain’ in the human body. The communication network between the gut and the CNS is very complex, including an intestinal nervous system branch, the sympathetic and parasympathetic nerve vegetative nervous system, and neural immune and neuroendocrine signal pathways (Refs 3,70,71). In terms of the gut microbiota’s impact on the brain, researchers have used different methods, including antibiotics, probiotic therapy, faecal microbiota transplantation (FMT), gastrointestinal infections and GF studies, to demonstrate its significant influence on mental health (Ref. 72). However, underlying mechanisms have not been fully elucidated. The gut microbiota is a key regulator of the MGB axis, which impacts on host physiological functions, emotional changes and social behaviours by regulating neural-, metabolic-, immune- and hormonal- (e.g., oestrogen) mediated mechanisms (Refs 70,73). In animal models and clinical studies, gut microbiota compositional changes were associated with brain function, e.g., FMT from patients with MDD induced depression-like behaviour in GF mice (Refs 74,75), indicating that intestinal microecological dysbiosis may occur before depression. Sanada et al. reported that abundance of the *Prevotellaceae* family and *Coprococcus* and *Fecalibacterium* genera was lower in patients with MDD when compared with non-depressed controls (Ref. 57). Additionally, Sovijit et al. found that progesterone in a PMD mouse model increased *Lactobacillus spp.* in the intestines, which improved depression- and anxiety-like behaviours (Ref. 76). These studies suggest that the MGB axis may have important roles in PMD, with axis imbalance triggering PMD mechanisms, potentially related to inflammation. Next, we elaborate on and summarize MGB axis mechanisms that mediate PMD occurrence and development from an inflammation perspective.

## Gut microbiota metabolite effects on PMD

### SCFAs effects on PMD

#### SCFAs

SCFAs are one of the most important end-products of intestinal microbial metabolism and are produced by the glycolysis and fermentation of indigestible carbohydrates (Ref. 77). Mainly found in the cecum and colon of animals and humans, SCFAs are widely distributed in enteroendocrine, immune and nerve cells (Refs 78,79). SCFAs are mainly generated by *Bifidobacterium spp.*, *Blautia hydrogentrophica*, *Prevotella spp.*, *Streptococcus spp.*, *Akkermansia muciniphilia*, *Bacteroides spp.*, *Anaerostipes spp.* and others (Ref. 80). Therefore, SCFAs content changes can indirectly reflect changes in intestinal microorganisms. SCFAs are mainly contain acetic acid (60%), propionic acid (20%), butyric acid (20%), etc. (Ref. 81). Mechanistically, SCFAs mainly act by binding to G protein-coupled receptors (GPCRs), mainly GPR43, GPR41 and GPR109A. Receptors are distributed in most human tissues; GPR41 is mainly distributed in adipose tissue, and GPR43 is highly expressed in immune cells, both of which bind to acetate, butyrate and propionate, while GPR109A is mainly expressed in adipose tissue and immune cells, and is only be activated by butyrate (Ref. 82).

#### SCFAs mechanisms in PMD

**Figure 3.**
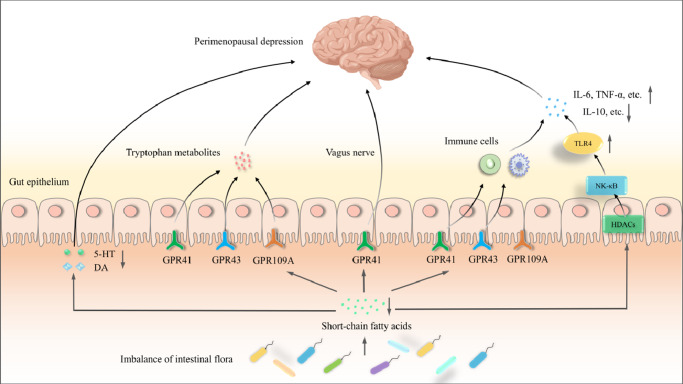
Possible SCFAs-mediated mechanisms in perimenopausal depression. In the vagus nerve pathway, SCFAs mainly bind to GPR41, which is highly expressed in the sympathetic nervous system, thus inhibiting the vagus nerve, which is widely distributed in the intestine. In the immune pathway, SCFAs inhibit HDACs, inhibit NF-κB and TLR4 activation, reduce proinflammatory factor levels (IL-6, TNF-α, etc.), and increase anti-inflammatory factor levels (IL-10, etc.), thus exerting antidepressant roles (Refs 98,99). In the neuroendocrine pathway, SCFAs affect the central nervous system by promoting neurotransmitter secretion, such as 5-HT and DA. In the metabolic pathway, SCFAs exert neuroactive effects by binding to three GPCRs (90). Thus, in a dysregulated gut microbiota, SCFAs content decreases, which inhibits the aforementioned effects and eventually leads to perimenopausal depression. SCFAs: short-chain fatty acids; NF-κB: nuclear transcription factor-κB; 5-HT: 5-hydroxytryptamine; HDACs: histone deacetylase; TNF-α: tumor necrosis factor alpha; GPCR: G protein-coupled receptor; TLR4: toll-like receptor 4.

SCFAs are important mediators in the MGB axis, with crucial roles in the neurobiological mechanisms underlying depression. They directly or indirectly participate in vagal, immune, neuroendocrine and metabolic pathway regulation in the MGB axis (Ref. 77) (Figure 3). GPCRs in intestinal epithelial and immune cells modulate inflammatory responses induced by SCFAs activation (Ref. 31). Studies have indicated that SCFAs activate GPR43 (i.e., free fatty acid receptor 2, FFAR2) and GPR41 (FFAR3), promote peripheral macrophage, dendritic and T cell activation to exert immune effects, affect immune regulatory T (Treg) cell proliferation and development and inflammatory mediator recruitment, and increase anti-inflammatory factor expression. Thus, SCFAs reduce peripheral inflammatory factor damage to the brain and exert antidepressant roles (Refs 83,84,85). Butyrate reduces lipopolysaccharide (LPS)-induced NF-κB activation via GPR109A (Ref. 86). Acetate binding to GPR43 activates NLRP3 inflammasome activation in the colon (Ref. 87). Additionally, intestinal 5-HT levels decrease upon SCFAs depletion, while acetate, butyrate and isobutyrate indirectly promote 5-HT production.

Considerable evidence now suggests that SCFAs are implicated in depression development. In Polish women (aged approximately 50 years old) with depression, acetic acid and isocaproic acid levels were lower in depressed women compared to healthy control women, while acetic acid and propionic acid concentrations were negatively correlated with depression scores (Ref. 88), thereby indicating that circulating SCFAs levels could be used as depression severity indicators. Animal studies reported that increasing the intestinal SCFAs content, particularly propionate and butyrate, effectively improved menopausal symptoms in OVX rats. Notably, propionate stimulated *Bifidobacterium*, thereby maintaining intestinal barrier permeability, while decreased levels putatively dysregulated neurotransmitter signalling, inflammation and oxidative stress (Ref. 88). Li et al. reported that intrarectal sodium propionate administration for 1 week in chronic unpredictable mild stress (CUMS) rats upregulated 5-HT, norepinephrine (NE) and dopamine (DA) levels, and improved intestinal microecological balance to alleviate depression-like behaviour (Ref. 89). Additionally, butyrate also exerted anti-inflammatory and protective intestinal barrier effects, with potential effects in the immune system and toward ischemic injury. For example, in a dynamic alteration study of the gut microbiota (Ref. 90), the butyrate-producing gut microbiota *Eubacterium* was found to be significantly reduced in a group of PMD model mice, while the relative abundance of gut microbial species, such as *Escherichia coli* and *Veillonella*, increased over time after surgery in OVX mice. Significant differences in gut microbial species abundance were recorded between OVX and control groups at different time points. Thus, *Veillonella* and *E. coli* may represent intestinal pathogens that affect the immune system and cause inflammatory responses. Notably, *Veillonella* is capable of producing acetate and propionate, thereby maintaining homeostasis in humans (Ref. 91). However, it has also been found that *Veillonella* can activate macrophages through the LPS/TLR4 pathway, triggering intestinal inflammation and further aggravating intestinal flora imbalance (Ref. 92). These findings suggest that oestrogen could reduce intestinal inflammation by regulating the abundance of butyrate-producing gut microbiota species. Thus, SCFAs may play important roles in PMD via the MGB axis.

Current studies report that ketamine exerts anti-inflammatory and neurotransmitter effects, and effectively improves treatment-resistant depression (Ref. 93). Ketamine is an N-methyl-D-aspartate receptor (NMDAR) antagonist, which alters intestinal microbiota composition (Ref. 94). It was reported that ketamine significantly increased *Lactobacillus johnsonii* levels in LPS-induced depressed mice (Ref. 95). Additionally, ketamine and its metabolites improved SCFAs-producing microbiota, such as *Butyricimonas*, *Turicibacter*, *Clostridiales*, etc., thereby improving the depression state (Ref. 95). However, while the impact of ketamine on gut microbiota composition has been documented to some degree, how ketamine regulates the intestinal microbiota and its metabolites, such as SCFAs metabolism, remains unclear. Therefore, further studies should investigate the mechanisms whereby ketamine, as a promising antidepressant, regulates gut microbiota metabolism and associated metabolites. The α-amino-3-hydroxy-5-methyl-4-isoxazolepropionic acid (AMPA) receptor-brain-derived neurotrophic factor (BDNF) mechanistic target of rapamycin (mTOR) signalling pathway is postulated to enhance synaptic function in the medial prefrontal cortex and contribute to rapid antidepressant ketamine effects (Ref. 96). Additionally, studies have also shown that women are more sensitive to ketamine than men (Ref. 97). However, the role of ketamine in PMD via the MGB axis remains unclear; therefore, further studies are required to clarify this.

### Monoamine neurotransmitters

The central monoamine neurotransmitter hypothesis posits that positive pleasure and happiness emotions are related to monoamine neurotransmitters in the brain, while a deficiency of 5-HT, NE, DA and other neurotransmitters is implicated in depression onset. Importantly, the development of first and second-generation antidepressants was based on this hypothesis (Ref. 101). Some researchers postulated a ‘three primary colour model of emotion,’ suggesting that NE was related to stress, DA to happiness, and 5-HT to depression (Ref. 3). Current studies have reported that female depression caused by oestrogen deficiency may be related to 5-HT deficiency (3). Estradiol (E_2_) has important roles regulating 5-HT synthesis, increasing 5-HT receptor 2A (5-HT2A) expression and reducing 5-HT catabolism (Ref. 102). These effects may be mediated by E_2_ binding to intracellular ER, where ER interacts with oestrogen response elements (tryptophan hydroxylase 2 (TPH2), serotonin transporter (SERT) and monoamine oxidase-A (MAO-A) in target gene promoter sequences (Refs 102,103,104). High E_2_ levels can inhibit MAO activity, thereby slowing monoamine neurotransmitter degradation and maintaining 5-HT concentrations at normal levels. Studies have shown that MAO-A levels in perimenopausal women are higher than those in premenopausal women, suggesting that levels are potentially related to changes in female sex hormones (Ref. 105). Animal studies have suggested that female rats have higher 5-HT or 5-hydroxyindoleacetic acid (5-HIAA) concentrations in the whole brain, forebrain, raphe, frontal cortex, hypothalamus and hippocampus when compared to male rats (Ref. 106). Tian et al. reported that when compared to a normal group, depression-like behaviours in CUMS rats were significantly increased, 5-HT, 5-HIAA, 5-HT/5-HIAA and TPH2 protein expression in the hippocampus were decreased, and SERT and MAO-A protein expression were increased (Ref. 107). Xiao et al., in their randomized controlled study, observed that improved depression-like behaviours in rats were associated with increased DA, 5-HT and NE levels in the hippocampus and serum (Ref. 108). Another study reported that 5-HT levels in the hippocampus and amygdala were lower in PMD model rats treated with 4-vinylcyclohexene dicyclic oxide when compared to control rats (Ref. 109). Zhang et al. indicated that depression-like behaviours in perimenopausal mice were related to increased 5-HT levels, and that the mechanism was possibly related to enhanced TPH2 expression (Ref. 110). This evidence suggests that neurotransmitters have important roles in PMD, but unfortunately, one-third of patients with MDD do not respond to current antidepressants (Ref. 111).

#### Monoamine neurotransmitters and the MGB axis

Intestinal epithelial cells have endocrine and paracrine functions, thus affecting neurotransmitter production and associated precursors. The gut microbiota synthesizes neurotransmitters, e.g., *Lactobacillus spp.* and *Bifidobacterium spp.* produce γ-aminobutyric acid (GABA), *Escherichia spp.*, *Candida spp.* and *Enterococcus spp.* produce 5-HT, *Bacillus spp.* produce DA, *Lactobacillus spp.* produce acetylcholine, and *Bacillus spp.* and *Saccharomyces spp.* produce NE (Refs 33,112). These molecules are not only involved in communications between the gut microbiota, but also in systemic and peripheral effects that affect brain function. More than 90% of 5-HT is produced in the human gut (Ref. 113). The intestinal microbiome electrically stimulates the vagus nerve, thereby altering neurotransmitter concentrations, such as 5-HT, glutamate and GABA in rodent and human brains (Ref. 100). Approximately 90% of peripheral 5-HT is produced by enterochromaffin cells, and 5-HT is synthesized from tryptophan (TRP) via the TRP hydroxylase 1 pathway. However, TRP metabolism also involves another pathway, the kynurenine (KYN) pathway. In inflammatory states, TRP metabolism is biased toward the KYN pathway, resulting in decreased 5-HT synthesis and increased KYN and associated metabolite levels, such as quinolinic acid. These metabolites are neurotoxic, activate NMDA receptors, and increase glutamatergic neurotransmission, thereby exacerbating neuroinflammation (Ref. 29). Also, neuroinflammation reduces 5-HT synthesis by activating the TRP metabolic pathway, leading to decreased 5-HT levels (Ref. 29). 5-HT regulates neuroinflammation via its receptors, such as 5-HT1A and 5-HT2A. For example, 5-HT1A receptor activation inhibits microglia overactivation and reduces proinflammatory cytokine release (Ref. 29). Another study reported that 5-HT binding to 5-HT receptors on microglia induced the release of cytokine-bearing exosomes, providing an alternative mechanism for regulated gut-induced neuroinflammation (Ref. 114). Ma et al. observed that when compared to female CUMS mice, *TPH2* knockout CUMS mice had significantly lower 5-HT serum levels and worse cognitive dysfunction, and also autophagy levels in the hippocampus were increased, neuroinflammatory responses were increased, and gut microbiota disorders were recorded, and mouse cognitive impairment was significantly improved (Ref. 115). Huang et al. reported that *Bifidobacterium infantis* increased TRP levels in plasma from rats, decreased 5-HT levels in the frontal cortex and dopamine metabolites in the cortex, and thus improved depressive symptoms (Ref. 116). Another study reported that increased TPH2 and 5-HT expression in the hippocampus and intestinal tissues of CUMS mice was associated with improved depression-like behaviours and gastrointestinal dysfunction (Ref. 117). Thus, neurotransmitters produced directly or indirectly by the gut bacteria may have important roles in PMD by binding to specific CNS receptors.

## Interactions between oestrogen and the gut microbiota

The gut microbiota has important roles in the female reproductive endocrine system by interacting with oestrogen, androgens, insulin and other hormones (Ref. 118). Of these, oestrogen deficiency is an important influencing factor in PMD. Oestrogen is a steroid hormone that is mainly secreted by the ovaries and the placenta during pregnancy (Ref. 119). E_2_ is the most biologically active oestrogen in women, and the main oestrogen that affects the main brain functions (Ref. 119). Oestrogen in the blood has both free and conjugated forms, of which the latter is the main form, but the biologically active the former (Ref. 119). Oestrogen is a major gut microbiome regulator, with the gut microbiome gene pool (‘oestrogen-ome’) capable of metabolizing oestrogen (Ref. 120). The hormone not only prevents beneficial bacterial loss and promotes their growth and reproduction, but also reduces pathogenic bacterial numbers and reduces lipopolysaccharide (LPS)-induced inflammation, thus exerting anti-inflammatory roles (Ref. 81). Gut microbiota dysbiosis in middle-aged women may potentially reduce free oestrogen levels and trigger oestrogen-related pathology, leading to depressive disorder moods. In terms of oestrogen interactions with gut microbes, the vast majority of studies have been conducted in animal models (Refs 121,122). For example, Li et al. showed that 3β-hydroxysteroid dehydrogenase, expressed by *Klebsiella aerogenes*, effectively degraded E_2_, thereby reducing levels in the mouse brain and blood, and eventually leading to depression-like behaviours (Ref. 123). Another study observed that *Proteobacteria* and LPS biosynthesis were reduced in male mice and also in a PMD mouse model treated with E_2_, indicating that oestrogen reduced intestinal permeability and LPS-induced inflammation, thereby reducing metabolic endotoxemia (Ref. 60).

**Figure 4.**
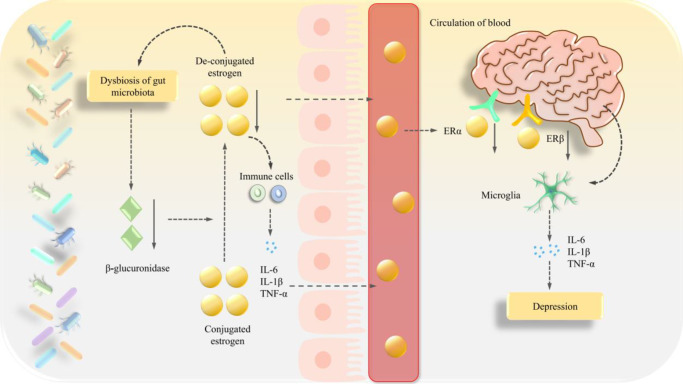
Potential interaction mechanisms between gut microbiota and oestrogen in perimenopausal depression (PMD). Gut microbiota dysbiosis reduces β-glucuronidase production, impairing the deconjugation of conjugated oestrogen into bioactive free oestrogen. Oestrogen deficiency promotes proinflammatory cytokine secretion (e.g., TNF-α, IL-1β, IL-6) by T cells and macrophages, leading to peripheral immune activation and chronic inflammation. These peripheral cytokines traverse the gut-brain axis, activate microglia and amplify neuroinflammation through sustained release of proinflammatory mediators. Concurrently, diminished free oestrogen entry into the brain suppresses oestrogen receptor β (ERβ) signalling, exacerbating depressive phenotypes in PMD. Furthermore, oestrogen deficiency perpetuates gut dysbiosis, establishing a vicious cycle between neuroendocrine dysfunction and microbial imbalance.

Only a few clinically relevant studies in this area have been published; Shin et al. divided subjects based on low, medium and high sex hormone levels, and showed that females in the high-dose group had increased *Bacteroidetes* and decreased *Firmicutes* abundance when compared with females in the low-dose group, while *Slackia* and *Butyricimonas* were significantly negatively correlated with serum estradiol levels (Ref. 124). In a paired premenopausal and postmenopausal female study, Santos-Marcos et al. reported that serum estradiol levels were positively correlated with the *Gammaproteobacteria* class and an unknown genus from *Myxococcales*, which was negatively correlated with *Prevotellaceae* (Ref. 36). Zhu et al. identified a weak positive correlation between estradiol levels and *Shewanella putrefaciens* and *Erwinia amylovora* (Ref. 125). These results suggest that interactions between the gut microbiota and oestrogen have key roles in PMD development via immune-mediated inflammatory pathways (Figure 4).

## Estrogen and inflammatory responses

Many studies have shown that depression is a neuroinflammatory disease, with neuroinflammation implicated in depression occurrence and development (Ref. 126). Studies have also shown that decreased ovarian function in women during menopause is associated with increased proinflammatory cytokine levels (Ref. 127). E_2_ exerts regulatory effects on NF-κB, which is a central regulator of inflammatory pathways and regulates multiple target gene expression. Oestrogen deficiency affects oestrogen target gene expression, leading to increased IL-7 levels and promoting T cell activation, which further induces proinflammatory cytokine secretion, such as IL-1, IL-6 and TNF (Ref. 128). Currently, considerable evidence suggests that oestrogen exerts anti-inflammatory effects by binding to the ER. The oestrogen receptors ERα, ERβ and GRP30 are widely expressed in microglia, astrocytes and neurons, and exert anti-inflammatory effects (Refs 129,130,131,132,133,134). Upon binding to the ERα in microglia, E_2_ activates phosphatidylinositol 3-kinase (PI3K)/protein kinase B (AKT) signalling, which in turn inhibits NF-κB activation, thereby reducing proinflammatory cytokine transcription (Refs 135,136). E_2_ also increases GABA levels in the hippocampus and frontal cortex by activating ERβ and/or GPR30, and up-regulates GABA-related genes in the amygdala and hippocampus (Ref. 137). Studies have shown that increasing ERβ expression alleviates depression-like behaviours during perimenopausal periods in OVX-CUMS mice (Ref. 110), whereas *ERβ* knockout mice have significantly increased anxiety-like behaviours (Ref. 138). Furukawa et al. found that E_2_ may affect social behaviour in OVX mice by regulating TPH expression in the raphe nucleus and serotonin release in the amygdala via GPR30 (Ref. 139). Upon activation, microglia released proinflammatory cytokines (TNF-α, IL-1β and IL-6), which initiated neuroinflammation (Ref. 140). Also, neurons triggered ATP release by activating NMDARs, which induced microglial processes (Ref. 141) and led to depression (Refs 142,143). Clinical studies also reported that peripheral estradiol serum levels in perimenopausal women were inversely correlated to serum IL-8 and TNF-α levels, and also microglial and astrocyte reactivity (Ref. 96). Thus, oestrogen may exert anti-inflammatory effects by activating the ER on glial cells, while its deficiency exerts inflammatory responses in glial cells, which impacts on brain function. Notably, there is a ‘critical period’ for oestrogen neuroprotective effects. It was reported that oestrogen therapy should be given immediately after brain injury, as administration at 122 weeks after oophorectomy results in treatment ineffectiveness, and long-term oestrogen deprivation decreases hippocampal ERα receptors (Ref. 132). Additionally, oestrogen, above physiological doses, was shown to activate inhibitory ERβ levels, thereby aggravating depression-like behaviours (Refs 144,145). Furthermore, several studies reported individual efficacious differences for oestrogen alone in treating PMD, e.g., transdermal oestrogen (0.1 mg/day for 4 or 12 weeks) or oral oestrogen (1.25 mg/day for 4 weeks) improved symptoms in patients with PMD (Ref. 146). Joffe et al. observed that perimenopausal women with depression had improved symptoms after transdermal estradiol treatment (0.05 mg/day for 8 weeks) (Ref. 147). However, other studies reported no significant differences from placebo (Ref. 148). Therefore, oestrogen therapy for PMD has limitations. In the future, multi-centre, large-sample, long-term randomized controlled trials must systematically evaluate dose–response relationships, intervention times and biomarker safety to clarify the clinical applicability of oestrogen therapy.

## The gut microbiota and inflammatory responses

Recent studies have shown that the gut microbiota has an increasingly close relationship with inflammatory responses in host physiological and pathological processes. Intestinal microbes maintain mucosal barrier integrity by regulating intestinal epithelial cell growth and differentiation, tight junction protein expression and intestinal mucosal permeability. Changes in intestinal microbial composition can damage the intestinal mucosal barrier, destroying connection functions between cells, increasing permeability and increasing the transport of inflammatory mediators (Ref. 31). Inflammatory responses associated with gut microbes are not only present in gastrointestinal diseases, such as irritable bowel syndrome and colorectal cancer, but also affect cardiovascular, reproductive, metabolic, autoimmune and neurodegenerative diseases (Ref. 31). A potential link between the gut microbiota and MDD is low-grade chronic inflammation (Ref. 81). Studies have shown that gut microbiota metabolites and microbial cell components (e.g., LPS) can pass through the damaged intestinal barrier (‘intestinal leakage’), leading to increased inflammatory factor levels, such as IL-6, TNF-α and IL-1β, thereby exacerbating systemic inflammatory responses (Refs 33,149,150). These cytokines can reach the brain via neuroanatomical and neuroendocrine pathways and influence mental health and behaviour (Ref. 151). Oestrogen fluctuations in perimenopausal women can aggravate gut microbiota imbalance, which then triggers the activation of the following key inflammatory signalling pathways.

### The TLR4/NF-κB pathway: the endotoxin-driven inflammatory cascade

Oestrogen regulates tight junction protein expression (e.g., occludin, claudin-5 and ZO-1) by activating oestrogen receptors in intestinal epithelial cells, thereby reducing intestinal mucosal permeability and preventing bacterial products (e.g., LPS) from entering the circulation. However, when oestrogen is deficient, gut microbiota dysbiosis occurs and is characterized by relative decreases and increases in *Firmicutes* and *Bacteroidetes*, respectively, which in turn promote increased Gram-negative bacteria levels, such as *Desulfurvibrio*, and also increased LPS release (Ref. 152). LPS enters the blood circulation via an impaired intestinal barrier and binds to TLR4 on macrophage surfaces. Upon TLR4 activation, the myeloid differentiation factor 88 (MyD88)-dependent pathway recruits IL-1 receptor-associated kinase, activates TRAF6, and then phosphorylates IκB kinase (IKK). IKK then promotes NF-κB inhibitor protein (IκB) degradation, releases NF-κB (p50/p65 dimer) into the nucleus and induces proinflammatory factor release (e.g., IL-1β, TNF-α and IL-6) to trigger peripheral inflammation. These circulating inflammatory factors enter the brain via weak areas in the BBB, such as the thalamus and hippocampus, and activate TLR4 in microglia. This activation drives microglia transformation to a proinflammatory phenotype (M1), releasing reactive oxygen species (ROS), IL-6 and TNF-α, and inhibiting neurogenesis, which in turn initiates neuroinflammation (Ref. 4). Oestrogen inhibits TLR4/NF-κB pathways in macrophages in the intestinal lamina propria by binding to oestrogen receptors and reducing proinflammatory factor release (e.g., IL-6 and TNF-α). Over-activated TLR4 signalling in perimenopausal women, due to decreased oestrogen levels, promotes peripheral and central neuroinflammation (such as increased hippocampal IL-1β levels), exacerbates synaptic plasticity damage and ultimately triggers depression (Ref. 153). Studies have shown that probiotics inhibit the NF-κB pathway by stabilizing IκBα, thereby reducing proinflammatory cytokine production (Ref. 153,154). For example, *Lactobacillus fermentum CQPC04* inhibited NF-κBp65 activation in the colonic tissues of mice with colitis, thereby reducing intestinal inflammation, but these inhibitory effects were enhanced by increasing doses of *L. fermentum CQPC04* (Ref. 155). Therefore, the gut microbiota appears to have important roles in PMD by regulating TLR4/NF-κB signalling.

### The NOD-like receptor protein 3 (NLRP3) inflammasome pathway

Inflammasomes play a key role in the activation of the innate immune system and the maturation of inflammatory cytokines. The dysregulation of inflammasomes may be related to MDD (Ref. 156). Clinical studies have shown that treatment failures in patients with major depression are associated with elevated inflammatory mediator serum levels (Ref. 111). In recent years, researchers have proposed the ‘microbiota-gut-inflammasome-brain axis,’ which suggests that interactions between the intestinal flora and inflammasomes can affect the intestinal microecological balance and physiological functions in the brain (Ref. 23). In particular, NLRP3 inflammasomes have important roles in depression occurrence and development. Studies have shown that Gram-negative bacteria, such as *Francisella novicida*, *Salmonella typhimurium*, *Citrobacter* and *E. coli*, can stimulate bone marrow-derived macrophages to produce IL-1β and IL-18 in an NLRP3-dependent manner (Ref. 157). An *Aspergillus* chimaera was shown to stimulate newly recruited monocytes to induce NLRP3-dependent IL-1β release and exacerbate intestinal inflammatory injury (Ref. 158). Recent evidence has also shown that the gut microbiota promotes proinflammatory cytokine production such as IL-1β via NLRP3 inflammasome activation, leading to acute pancreatitis, colitis and depression (Ref. 159). Together, these findings suggest that gut microbes activate the inflammasome in immune cells in an NLRP3-dependent manner.

#### The biological characteristics and activation mechanisms of the NLRP3 inflammasome

**Figure 5.**
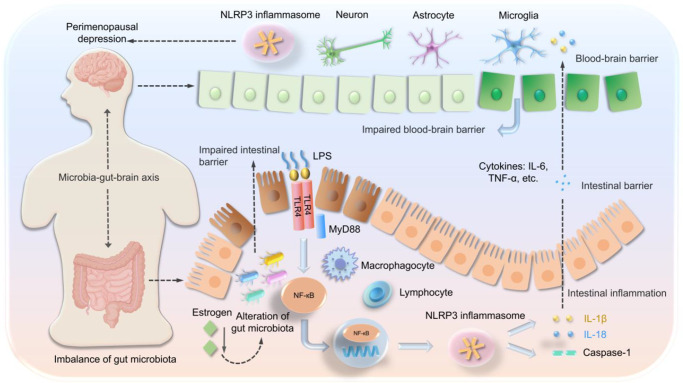
NLRP3 inflammasome mechanisms in PMD mediated by the MGB axis. Oestrogen deficiency disrupts the gut microbiota, which disrupts the gut barrier and leads to harmful substance release (e.g., LPS). TLR4 in intestinal epithelial and immune cells recognizes LPS and activates NF-κB and the NLRP3 inflammasome via MyD88 to promote caspase-1 maturation and inflammatory cytokine (IL-1β and IL-18) release, thereby destroying the intestinal barrier and increasing intestinal permeability, triggering intestinal and peripheral inflammation. Inflammation increases BBB permeability and enhances microglial activity via the MGB axis, leading to neuroinflammation and NLRP3 inflammasome activation, which ultimately causes PMD. TLR4: toll-like receptor 4; LPS: lipopolysaccharide; MyD88: myeloid differentiation primary response protein 88: NLRP3: NOD- like receptor protein 3; NF-κB: nuclear transcription factor-κB; IL-1β: interleukin-1β; and IL-18: interleukin-18; PMD: perimenopausal depression.

The NLRP3 inflammasome is a complex composed of NLRP3 receptor protein, adaptor protein apoptosis-associated speck-like protein containing a CARD (ASC) and Caspase-1, which is widely distributed in the immune system and non-immune systems, such as macrophages, B cells, T cells, neurons, astrocytes and microglia (Refs 31,160). The mechanism of action of NLRP3 inflammasome involves dual signalling activation: The first priming signal is the release of LPS due to dysbiosis of gut microbiota (such as a decreased F/B ratio) (Ref. 4). Pattern recognition receptors (PRRs), such as TLR4, are activated upon recognizing LPS, subsequently inducing activation of the NF-κB signalling pathway (Ref. 4). NF-κB translocates to the nucleus, upregulating the transcription and expression of precursor proteins such as NLRP3, pro-IL-1β and pro-IL-18 (Ref. 4). The second activation signal occurs when gut microbiota metabolites (such as ATP and SCFAs) or pathogen-associated molecular patterns (PAMPs) trigger the binding of NLRP3 with ASC and Caspase-1, assembling into the inflammasome complex (Refs 4,156,161,162). Activated Caspase-1 cleaves pro-IL-1β and pro-IL-18 into mature IL-1β and IL-18, and induces Gasdermin d-mediated pyroptosis, releasing large amounts of IL-1β and IL-18 to further amplify the inflammatory response (Refs 4,156,161,162). IL-1β and IL-18 then activate microglia via an impaired BBB or vagal afferent signals. IL-1β activates microglia via IL-1R1 to release TNF-α and IL-6, and inhibits hippocampal neurogenesis (Figure 5).

#### NLRP3 activation and neuroinflammatory injury in the hippocampus

The hippocampus is an important part of the limbic system. The NLRP3 inflammasome and its activation in the hippocampus were related to depression-like behaviour induced by oestrogen deficiency in animals. Several animal studies reported that depression-like behaviour in OVX animals was associated with NLRP3 inflammasome activation and enhanced IL-1β, IL-18, TLR4 and NF-κB expression in the hippocampus (Refs 153,163,164,165). However, *IL-1β, IL-18, caspase-1* and *NLRP3* inflammasome gene knockouts improved depression-like behaviour in mice (Refs 4,166). Clinical studies also reported that serum IL-1β, IL-18, NLRP3 and caspase-1 expression in MDD patients was significantly increased, but after antidepressant treatment, indicators were significantly decreased, thereby effectively improving depressive states in patients (Refs 167,168). Therefore, these observations reinforce the notion that the NLRP3 inflammasome may be at the centre of inflammatory cascades following oestrogen deficiency, ultimately leading to depression. Wong et al. showed that *caspase-1* knockout reduced anxiety- and depression-like behaviours in mice with chronic restraint stress (CRS), and found that the antibiotic minocycline regulated the gut microbiota and inhibited caspase-1, thereby improving depression-like behaviour and gut microbiota disorder in CRS mice (Ref. 156). Zhang et al. observed that behavioural changes were improved in *NLRP3* gene-deficient mice when compared to CUMS mice, and when faecal microbes from *NLRP3* gene-deficient mice were transplanted into recipient CUMS mice, mice exhibited significantly improved depression-like behaviour (Ref. 169). Another study confirmed that NLRP3 inflammasome activity in CUMS mice was inhibited by a TLR4 inhibitor, thus effectively relieving a depressive state in animals (Ref. 170). Studies have shown that chronic ethanol exposure (CEE) induces depression-like behaviour in mice via FMT. Severe hippocampal neuroinflammation and NLRP3 inflammasome activation are also observed in recipient mice, indicating that depression-like behaviour is regulated by the gut microbiota (Ref. 171). Inflammatory factors are critical for NLRP3 inflammasome activation in the hippocampus. A causal mediation analysis in mice receiving the NLRP3-shRNA group FMT showed increased *Firmicutes*, *Actinobacteria*, *Erysipelotrichi* and *Allobaculum* abundance, and reduced *Bacteroidetes*, *Bacteroidia*, *Verrucomicrobiae* and *Bacteroidales* abundance, while mice showed increased depression-like behaviour risks, which were mediated by LPS, IL-1β, TNF-α, interferon (IFN)-γ and IL-12p70 (Ref. 171). These observations suggest that the gut microbiota activates the NLRP3 inflammasome in the hippocampus via peripheral inflammatory factors (Ref. 171). These aforementioned studies suggest that the MGB axis-mediated NLRP3 inflammasome in the hippocampus may be an important treatment target for PMD. However, the interplay between the gut microbiota and the NLRP3 inflammasome is poorly understood. Notably, most current studies have focused on animal models, while humans and rodents exhibit significant differences in NLRP3 expression profiles: the activation threshold of NLRP3 in human microglia is lower than that in rodents, and human microglia are more sensitive to oestrogen fluctuations (Ref. 172), suggesting that species-specific mechanisms require further validation.

### The JAK–STAT pathway

As an important inflammatory regulatory mechanism, the Janus kinase-signal transducer and activator of transcription (JAK–STAT) signalling has important roles in the pathological mechanisms underlying depression (Ref. 173). This pathway promotes microglia activation and inflammatory factor release, which in turn affects neuronal function and synaptic plasticity, leading to neuroinflammation (Ref. 174). Similarly, the pathway has crucial roles in regulating intestinal inflammation and maintaining intestinal homeostasis (Ref. 175). Studies have shown that butyrate reduces inflammation by inhibiting histone deacetylase (HDAC) activity and down-regulating signal transducer and activator of transcription 3 (STAT3) phosphorylation (Ref. 31). Another study showed that butyrate-producing *F. prausnitzii* inhibited pathway activation by promoting expression of histone acetylation-mediated suppressor of cytokine signalling 1, thus exerting antitumor effects (Ref. 176). Recent studies also reported that the pathway mediated proinflammatory cytokine production and microglia proliferation, resulting in hippocampal synaptic deficits in LPS-induced and chronic social defeat stress (CSDS)-induced depression in mice, while tofacitinib (JAK inhibitor) administration attenuated depression-like behaviours in animal models (Ref. 177). Additionally, Sulkowska et al. observed that ERα activated the STAT3 pathway by activating JAK2 and SRC protein activity (Ref. 178). Thus, JAK–STAT signalling has important roles in depression; however, its role in PMD has not been reported.

One possible JAK–STAT pathway action mechanism is that LPS activates intestinal immune cells (e.g., macrophages) via TLR4, releasing IL-6, IFN-γ and TNF-α, which enter the CNS through the circulation and bind to cell surface receptors to activate JAK–STAT signalling. IL-6 binds the IL-6R/gp130 complex, and IFN-γ binds IFN-γR to activate receptor-associated JAK kinases (e.g., JAK1 and JAK2). JAK further phosphorylates STAT proteins (e.g., STAT3) to form dimers that enter the nucleus and induce indoleamine 2, 3-dioxygenase (IDO) expression. IDO then converts TRP to Kyn, which is further metabolized to neurotoxic quinolinic acid (QUIN) that activates NMDAR and induces glutamate excitotoxicity. At the same time, QUIN inhibits 5-HT synthesis. Additionally, JAK–STAT pathway activation drives microglial transformation to a proinflammatory phenotype (M1 phenotype), releasing IL-1β, ROS and activating other signalling pathways, thus contributing to depression (Ref. 179). Notably, oestrogen inhibits JAK2 phosphorylation and reduces STAT3 nuclear translocation by binding to ERβ (Ref. 180). However, during perimenopause, oestrogen levels are decreased and the inhibitory effects on JAK–STAT signalling are relieved, leading to increased IDO activity and 5-HT depletion, which may aggravate depressive symptoms.

## Potential gut microbiota applications for PMD treatment

**Figure 6.**
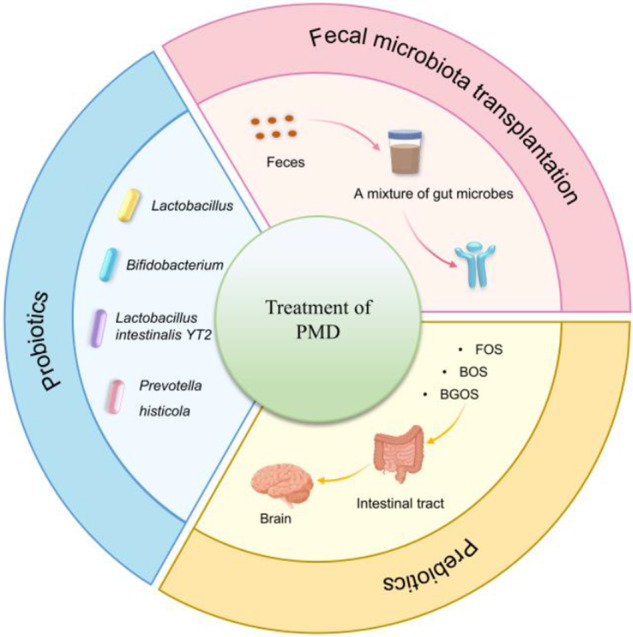
Potential gut microbiota application for PMD: therapeutic approaches targeting the microbiome, include FMT, probiotics (*Lactobacillus*, *Bifidobacterium*, etc.) and prebiotics (fructo-oligosaccharides, BOS, etc.). PMD: perimenopausal depression; FMT: faecal microbiota transplantation.

A growing body of evidence now suggests that different treatments targeting the microbiota can effectively improve depression by re-establishing the correct intestinal microecological balance. Among these, FMT, probiotics and prebiotics have potential clinical applications (Figure 6).

### FMT

FMT rapidly reshaped the gut microbiome by transplanting faecal microbiota from healthy donors into the gut of patients. FMT has been shown to relieve depressive state (Table 3), e.g., animal studies have shown that FMT from healthy donors alleviated alcohol-induced depression-like behaviour in mice (Ref. 96), and also improved stress-induced depression-like behaviour in rats by improving the gut microbial balance, reducing intestinal barrier damage and inhibiting neuroinflammation (Ref. 97). In a randomized controlled trial, two female patients with MDD, aged 50–60 years old, showed significant improvement in depressive symptoms after the oral administration of frozen FMT capsules for 4 weeks, and in one patient, effects lasted 8 weeks (Ref. 181). Furthermore, a recent randomized controlled, double-blind trial validated the feasibility, efficacy and safety of an FMT enema in adults with moderate to severe depression (Ref. 182). At the same time, despite potential side effects and complications, the accessibility and tolerability of enema is high in some patients, suggesting its great therapeutic potential (Ref. 182). Critically, FMT opens up new avenues for PMD treatment; however, there is a lack of clinical research on FMT in PMD, and more studies are required to fully verify its efficacy.

**Table 3.** FMT action mechanisms in depression

Study Model/Population	FMT Intervention	Main Outcomes	Action mechanisms	References
CEE Mouse Model	Gut microbiota from control and CEE mice were transplanted into recipient mice.	Recipient mice exhibited depression-like behaviours and hippocampal neuroinflammation. FMT reduced gut microbiota diversity, impaired gut barrier function and elevated inflammatory cytokine (IL–1β, TNF-α, IFN-γ) serum levels.	An imbalanced gut microbiota increased peripheral inflammatory factors, activated NLRP3 inflammasomes in the hippocampus, and then caused depression-like behaviours in mice. NLRP3-mediated neuroinflammation may have important roles in this process.	(Ref. 171)
Germ-Free Mouse Model	Microbiota from MDD patients was transplanted into germ-free mice.	Recipient mice exhibited depression-like behaviours. *Actinobacteria* levels increased, while *Bacteroidetes* levels decreased. Key carbohydrate metabolite levels (e.g., α-glucose, β-glucose, fructose, and succinate) increased, whereas glutamate, phenylalanine and related metabolites decreased.	The gut microbiota modulated depression-like behaviours via host metabolic pathways, particularly carbohydrate and amino acid metabolism. Metabolomics showed abnormal carbohydrate and amino acid metabolic pathways in the gut and serum.	(Ref. 183)
SD Rat Model	Rats received FMT from healthy controls or MDD patients after antibiotic-induced microbiota depletion.	Rats receiving faecal microbiota from MDD patients exhibited depression-like behaviours, increased plasma kynurenine/tryptophan ratios, elevated CRP levels, increased gut transit times and reduced microbiota richness and α-diversity.	The gut microbiota influenced depression by modulating tryptophan metabolism, inflammatory responses and gut motility.	(Ref. 184)
CUMS Mouse Model	Faecal microbiota from CUMS mice and healthy controls were transplanted into recipient mice treated with antibiotics.	Recipient mice exhibited anxiety- and depression-like behaviours. Relative *Lactobacillus* abundance decreased, while *Akkermansia* abundance increased. TNF-α, IFN-γ and IDO1 protein levels were significantly increased in the hippocampus.	1. Inflammatory pathways: the gut microbiota affected hippocampal function by regulating inflammatory cytokines such as TNF-α and IFN-γ, which in turn affected anxiety and depression-like behaviours. 2. Tryptophan metabolism: increased IDO1 activity caused changes in the tryptophan metabolism pathway, which were possibly related to depression-like behaviours.	(Ref. 185)
Clinical Trial in Refractory IBS Patients	Healthy donor feces were transplanted into refractory IBS patients via colonoscopy.	In 10 patients, 6 showed clinical responses. Post-FMT, gut microbiota diversity increased significantly at 4 weeks, with responders showing higher diversity than non-responders. *Bifidobacterium* abundance was higher in effective donors. HAM-D scores significantly decreased, with improved depressive symptoms.	Donor faecal microbiota rich in *Bifidobacterium* may be a key factor for successful FMT, potentially alleviating symptoms by modulating microbiota diversity and SCFAs production. Specific targets and mechanisms remain unclear.	(Ref. 186)
Clinical Trial in Refractory IBS Patients	A faecal microbiota suspension from healthy donors was infused into the gut of refractory IBS patients.	IBS symptoms significantly improved at 1 and 3 months post-FMT. IBS-QOL and symptom scores (IBS-SSS and GSRS) decreased. HAMA and HAMD scores also decreased, suggesting relieved depression and anxiety symptoms.	FMT improved IBS symptoms by altering gut microbiota diversity (increased Shannon diversity index) and composition (e.g., increased *Verrucomicrobia* and *Archaea*, particularly *Akkermansia* and *Methanobrevibacter*). Specific targets and mechanisms remain unclear.	(Ref. 187)
Clinical Trial in MDD Patients	Two female MDD patients (aged 50–60) orally received frozen FMT capsules.	Depressive symptoms improved, with decreased HAMD scores and improved gastrointestinal symptoms. Patient 1: HAMD ↓57% (4 weeks), GSRS ↓71%; Patient 2: HAMD ↓68% (4 weeks), GSRS ↓10%. Patient 1 showed increased SCFAs-producing bacteria (*Faecalibacterium*) and microbiota diversity, while Patient 2 showed increased inflammation-related bacteria (*Flavonifractor*, *Streptococcus*) and calprotectin levels.	Patient 1: Increased SCFAs-producing bacteria may have alleviated depressive symptoms via anti-inflammatory and neuroregulatory effects. Patient 2: Increased inflammation-related microbiota (e.g., *Streptococcus*) may be associated with incomplete symptom improvement. Specific targets and mechanisms remain unclear.	(Ref. 181)

Abbreviations: CEE: conjugated equine oestrogens; CUMS: chronic unpredictable mild stress; FMT: faecal microbiota transplantation; IBS: irritable bowel syndrome; IDO1: indoleamine 2,3-dioxygenase 1; MDD: major depressive disorder; NLRP3: NOD-like receptor protein 3; SCFAs: short-chain fatty acids; SD: Sprague Dawley.

### Probiotics

Probiotics are living microorganisms that, when given in sufficient doses, exert beneficial effects on host health (Refs 188,189). Probiotic strains mainly include *Lactobacillus* and *Bifidobacterium* as well as some *Streptococcus* and *Enterococcus* strains (Ref. 188). In addition to their positive effects on the gut, probiotics contribute to concentration changes in brain neurotransmitters and proteins, decrease cortisol levels, and alter serum cytokine levels, leading to behavioural changes (Refs 190,191). A recent study reported that probiotics alleviated CEE-induced depression-like behaviour in mice (Ref. 171). Animal studies also showed that *Lactobacillus* supplementation increased oestrogen levels and alleviated diseases caused by decreased oestrogen levels (Refs 192,193,194). Ganesh et al. observed that oral *Lactobacillus reuteri* administration alleviated colitis in stressed mice and reduced LPS and IL-6 blood levels, thereby improving depression-like behaviour (Ref. 195). However, two studies reported that *Lactobacillus* ingestion caused depression and anhedonia-like phenotypes in animals, as well as social behaviour abnormalities (Refs 196,197). Thus, probiotics must be used with caution to exert efficient antidepressant effects. Additionally, *Prevotella histicola* and *Lactobacillus intestinalis* YT2 were shown to improve depression-like behaviour in OVX mice (Refs 42,45). Of these probiotics, *P. histicola* reduced intestinal inflammation by down-regulating inflammatory factors (IL-6, IL-8 and TNF-α) levels in the ileum and colon of OVX mice, and then reduced TLR4, Myd88, IL-6, IL-8 and TNF-α expression in the hippocampus, thereby exerting antidepressant actions (Ref. 45). This research shows that probiotics may be important targeted therapies for PMD.

*Akkermansia muciniphila* (AKK) is a new class of psychiatric probiotic. AKK and its metabolites can effectively improve neuropsychiatric disease symptoms, such as depression and anxiety, by restoring the intestinal microbiota, rebuilding intestinal mucosal barrier integrity, regulating host immunity, and modulating intestinal inflammation and neuroinflammation (Refs 198,199,200,201). AKK also improves chronic low-grade inflammation by reducing proinflammatory factor levels such as IL-6, increasing anti-inflammatory factors such as α-tocopherol, and reducing LPS binding protein (Refs 202,203). Liu et al. showed that AKK ameliorated colitis in *TLR4*-null mice by increasing RORγt^+^ Treg cell proportions and activating their immune responses (Ref. 204). Goo et al. observed that FMT from normal to *Fmr1* knockout mice increased intestinal AKK levels, improved autism-like behaviours, and alleviated cognitive deficits and social withdrawal symptoms in recipient mice (Ref. 205). AKK also exerted antidepressant effects in a CRS-induced mouse depression model, with effects associated with increased β-alanyl-3-methyl-L-histidine and edaravone levels (Ref. 201). Therefore, AKK may have good therapeutic potential for PMD.

### Prebiotics

Prebiotics are non-digestible polysaccharides, such as oligosaccharides, fructans (fructo-oligosaccharides (FOS), and inulin), and galacto-oligosaccharides (GOS) that are present in many natural products and dietary ingredients. They are selectively used by host microbes and may benefit host health (Ref. 189). As an energy source for intestinal microorganisms, prebiotics are essential for intestinal health and can stimulate the immune system and antagonize harmful intestinal bacteria (Ref. 188). A prebiotic intervention appeared to inhibit proinflammatory and neurotoxic signalling pathways and upregulate a neuroprotective microglial phenotype in an α-synuclein overexpression mouse model (Ref. 46). Currently, there is a lack of research on the psychophysiological effects of prebiotics. These include the soluble dietary fibres GOS and FOS, which act as nutritional sources for *Bifidobacterium* and *Lactobacillus*, stimulating their gut activity and reproduction. Studies have shown that FOS and GOS modulate BDNF and synapsin expression in rodent brains, thereby improving anxiety-like behaviours (Ref. 206). Savignac et al. observed that GOS exerted anti-inflammatory and anti-anxiety effects by inhibiting increased IL-1β and 5-HT2AR levels induced by LPS in mice (Ref. 207). These studies suggest that prebiotics may be important molecules for PMD treatment.

## Conclusions and outlook

Studies examining gut microbiota alterations in PMD patients are controversial. However, it cannot be denied that gut microbiota composition and metabolites in patients with depression are significantly different from healthy individuals, with gut microbiota status in patients with depression more often in a proinflammatory state. Many studies have reported that the gut microbiota regulates inflammatory signalling via the NLRP3 inflammasome in the MGB axis and then affects brain homeostasis. However, how the brain regulates intestinal inflammation via efferent pathways remains unclear. Therefore, an in-depth understanding of the relationships between the gut microbiota and the NLRP3 inflammasome, and the identification of related foods or probiotics that regulate intestinal microecological balance, can provide new treatment directions for PMD. To date, research on gut microbiota mechanisms in oestrogen-deficiency-induced depression has mainly focused on animal models, but there is a lack of relevant clinical research on the dynamic monitoring of gut microbiota changes in depression. It will be important to investigate the effects of oestrogen deficiency on the gut microbiota and its associated metabolic and immune diseases in the short term. Additionally, there is a relative lack of clinical research on PMD. In the future, for patients with PMD, we need to comprehensively examine gut microbiota changes, perform microbial-targeted therapies, dynamically monitor gut microbiota changes, and conduct long-term, follow-up prognosis studies.

Animal models of OVX undergo surgical removal of the ovaries, resulting in a dramatic decrease in hormone levels, and this change is significantly different from the course of natural menopause in humans. Natural menopause in humans is a gradual process, during which the body has a series of complex physiological regulatory mechanisms to adapt to the changes in hormone levels. However, the OVX model lacks this natural adaptive regulatory process. Furthermore, microbiota changes and neuroinflammatory responses during perimenopause in humans may be influenced by multiple factors, including lifestyle, dietary habits and genetic background, which are difficult to fully simulate in animal models. This may be an important reason for the difference in the incidence of depression between OVX animal models and the actual situation in humans. Oestrogen may improve female depression through two pathways of action. On the one hand, oestrogen can indirectly inhibit depression by interacting with gut microbiota/intestinal epithelial cells, regulating the composition and function of the intestinal microbial community, affecting the synthesis and metabolism of neurotransmitters and regulation of the immune system. On the other hand, oestrogen can directly act on the nervous system by interacting with oestrogen receptors in the brain to regulate the release of neurotransmitters and neuroendocrine function, thereby improving depressive symptoms. It is not clear whether the interaction pathway between oestrogen and gut microbiota/intestinal epithelial cells is the main pathway. Although these probiotics showed positive effects in OVX animal models, it is not sufficient to conclusively infer the dominance of this pathway in the treatment of depression in humans. Therefore, the interaction between oestrogen and gut microbiota/intestinal epithelial cells cannot be considered as the main way to treat female depression based on the results of animal experiments, and more in-depth research based on humans is needed in the future.

Although several studies have shown that regulating the gut microbiota can improve depressive symptoms, therapeutic effects can significantly vary between individuals, with such effects potentially due to multi-dimensional interactions in host genetics, strain functions and metabolic phenotypes. Many studies have only focused on common probiotics such as *Lactobacillus* and *Bifidobacterium*, while other potentially beneficial or harmful microbial communities are poorly studied. This narrow perspective may mean that some important microbial-host interaction mechanisms are inadvertently missed or ignored. Therefore, to overcome the limitations of OVX animal models, to systematically integrate human pathophysiological characteristics and clinical data, to improve the value of translational medicine, and formulate corresponding translational research strategies, ‘precision microbiota medicine’ must be promoted via integrated multi-omics, artificial intelligence predictions and dynamic monitoring technology to address both standardization and ethical challenges (Table 4).

**Table 4.** Strategies addressing future PMD translational research

Transformation Strategy	Methods	Advantages	Challenges	References
1. Dynamic Hormone-Microbiota Animal Models	- Simulate perimenopausal hormone fluctuations (pulsatile estradiol administration) in OVX models. - Transplant faecal microbiota from PMD patients into germ-free animals combined with hormonal interventions.	May accurately simulate human hormone fluctuations and microbiota interactions, potentially reducing bias in static models.	Complex procedures and high costs. Must optimize hormone dosing and microbiota transplantation protocols.	(Refs 208,209)
2. Longitudinal Multimodal Human Cohorts	- Track women during perimenopause (aged 45–55) for 3–5 years, and regularly collect data on hormones, microbiota, inflammatory markers and behavioural outcomes. - Stratify analyses based on hormone fluctuation patterns (e.g., fluctuating versus abrupt decline).	May capture dynamic pathological processes and distinguish PMD-specific mechanisms from confounding factors (e.g., aging).	Potentially high sample attrition rates and large sample sizes are required. The integration of multi-timepoint data is challenging.	(Refs 24,210)
3. Multi-Omics Dissection	- Integrate metagenomics (microbiota), metabolomics (SCFAs), epigenomics (oestrogen receptor methylation) and neuroimaging data. - Apply Mendelian Randomization to validate causality.	May reveal synergistic mechanisms underlying microbiota-metabolite-immune-neural circuits and guide target discovery.	High data heterogeneity exists. Standardized analysis workflows must be developed. Statistical power depends on sample size.	(Refs 211,212)
4. Precision Stratified Intervention Trials	- Stratify PMD patients based on baseline biomarkers (e.g., F/B ratios and IL–6 levels). - Develop microbiota-hormone combination therapies (e.g., probiotics + low-dose oestrogen).	May improve treatment response rates and reduce heterogeneity in clinical trials.	Need to validate the universality of stratification criteria. Long-term safety of combination therapies remains to be assessed.	(Refs 213,214)
5. Integrating Organoid and AI Technology	- Develop patient-derived gut-brain organoid co-culture systems to simulate neuroimmune interactions under hormone deficiency. - Use AI to predict high-risk populations for PMD (based on hormone, microbiota and inflammatory profiles).	Break through species limitations to elucidate human-specific mechanisms. Enable early warning and personalized interventions.	Immature organoid culture techniques. AI models rely on high-quality annotated data.	(Refs 215,216)

Abbreviations: PMD: perimenopausal depression; OVX: ovariectomized; SCFAs: short-chain fatty acids.
